# Focal Cortical Dysplasia Type II: Somatic Mutations, Molecular Mechanisms, and Integrative Multi‐Omics Framework

**DOI:** 10.1002/cns.71170

**Published:** 2026-09-23

**Authors:** Zesheng Li, Yihe Wang, Jianwei Shi, Yumin Luo, Yongzhi Shan, Guoguang Zhao

**Affiliations:** ^1^ Department of Neurosurgery, Xuanwu Hospital Capital Medical University Beijing China; ^2^ Clinical Research Center for Epilepsy Capital Medical University Beijing China; ^3^ China International Neuroscience Institute Beijing China

**Keywords:** epileptogenic network, focal cortical dysplasia, mTOR signaling, single‐cell genomics, somatic mutation

## Abstract

**Background:**

Focal cortical dysplasia Type II (FCD II) is a major cause of drug‐resistant epilepsy in children. Somatic mosaic variants affecting the PI3K–AKT–mTOR pathway are implicated in a substantial proportion of cases. The links between genotype, cellular pathology, epileptogenic networks, and clinical outcome remain incompletely defined.

**Methods:**

This review synthesizes evidence on somatic mTOR–pathway variants, lesion development, pathological cell populations, circuit mechanisms, single‐cell and spatial multi‐omics approaches in FCD II.

**Results:**

The developmental timing, lineage distribution, and local expansion of somatic variants influence lesion extent and phenotypic heterogeneity. Dysmorphic neurons may contribute to epileptogenicity through altered intrinsic excitability and synaptic function, whereas balloon cells may predominantly affect local circuits through secretory and microenvironmental mechanisms. mTOR hyperactivation may also alter interneuron development, subtype specification, and synaptic connectivity, thereby disrupting excitation‐inhibition balance. Single‐cell and spatial approaches, including RNA velocity, pseudotime analysis, chromatin‐age inference, and somatic genotyping, provide tools to resolve pathological cell states and lineage trajectories.

**Conclusions:**

FCD II should be considered a mosaic developmental disorder involving both cell‐autonomous and non‐cell‐autonomous mechanisms. Integrating somatic variant detection with cellular, spatial, electrophysiological, and surgical outcomes from matched patient tissue may enable biologically meaningful stratification and support the development of novel therapies.

## Graphical abstract

Focal cortical dysplasia Type II (FCD II) is a major cause of drug‐resistant epilepsy and is commonly associated with somatic brain mutations in the PI3K–AKT–mTOR pathway. The timing, cell lineage, and spatial distribution of somatic variants influence lesion formation. Dysmorphic neurons and balloon cells may disrupt local circuit function and epileptogenic networks. Integrating genetic, single‐cell, and spatial multi‐omics data with electrophysiology, cortical organoids, and mosaic animal models, neuropathology, and clinical information may advance our understanding of FCD II and enable more precise surgical and therapeutic strategies.

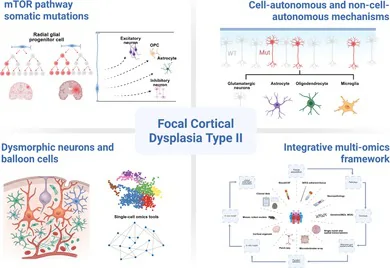

## 1 Introduction

Focal cortical dysplasia (FCD) is among the most common structural substrates of drug‐resistant epilepsy in children [1]. In pediatric cohorts undergoing surgical evaluation and resection, FCD is also one of the most frequent pathological diagnoses [2, 3]. Under the International League Against Epilepsy (ILAE) classification, FCD comprises types I–III. Mild malformation of cortical development (mMCD) and mild malformation with oligodendroglial hyperplasia in epilepsy (MOGHE) are related but distinct malformations of cortical development [4, 5, 6]. FCD Type II (FCD II) predominates in surgical series. It commonly presents with early‐onset, high‐frequency seizures and substantial neurocognitive and behavioral comorbidity, and frequently progresses to drug‐resistant epilepsy [1, 2, 7, 8]. Clarifying the molecular pathology of FCD II is therefore foundational for improving surgical stratification and identifying nonsurgical intervention targets.

Over the past decade, FCD II research has moved from radiological and pathological definition toward molecular stratification driven by somatic mutations. Somatic variants in the PI3K–AKT–mTOR axis explain a substantial fraction of cases. The mTOR‐related pathogenic mechanisms are increasingly traceable at single‐cell and spatial tissue resolution [9, 10, 11, 12]. The classical linkage between FCD morphology, PI3K–mTOR signaling, and therapeutic implications has been established [1]. How brain somatic mosaicism informs human lineage architecture and epileptic malformations of cortical development has been revealed [13]. The landscape of somatic activating and repressor variants in the mTOR pathway across cortical malformations was also studied [14]. Interneuron‐centered syntheses of mTORopathies have more recently broadened circuit‐level interpretation beyond excitatory dysplasia alone [15].

This review examines the developmental origins of FCD II and the contributions of dysmorphic neurons (DNs), balloon cells (BCs), interneurons, and glial‐vascular niches. It also considers how multi‐omics data may be integrated with clinical phenotyping. Our discussion centers on three main questions: (1) How do somatic mutations shape lesion extent and subtype? (2) How do mutant clones amplify hyperexcitability through cell‐autonomous, non‐cell‐autonomous, and circuit‐level mechanisms? (3) How can multi‐omics data be integrated into an actionable research framework? To improve interpretability, we stratify cited evidence using the four‐level framework summarized in Table 1.

**TABLE 1:** Categories of evidence used in this review.

Category	Description	Examples in FCD II
1. Direct human tissue	Observations in resected FCD II tissue without relying on trajectory algorithms	Histopathology (DNs, BCs); pS6 immunohistochemistry; bulk or single‐cell genotyping; spatial profiling; paired patch‐clamp when available
2. Experimental models	Mechanistic tests under defined genetic and developmental conditions	Mosaic rodent models; patient‐derived organoids; conditional pathway perturbations
3. Computational inference	Algorithm‐derived relationships that propose dynamics or ordering but do not by themselves prove lineage or causality	RNA velocity; pseudotime; potency scores; chromatin‐age models
4. Hypothesis	Explicit proposals awaiting prospective validation	Speculative IIa/IIb lineage frameworks; proposed spatial concordance between mosaic burden and SOZ/EZ

Abbreviations: BCs: balloon cells; DNs: dysmorphic neurons; EZ: epileptogenic zone; SOZ: seizure‐onset zone.

## 2 Clinical and Pathological Characteristics in FCD II

FCD histopathological classification has evolved from morphology alone toward multimodal integration [16]. Updated ILAE guidance emphasizes joint interpretation of imaging, pathology, and genetic evidence [1, 4]. Microscopically, FCD IIa is characterized by cortical lamination disorganization and DNs. FCD IIb adds BCs on the basis of FCD IIa. Whether IIa and IIb share a common ontogenetic path remains debated. DNs and BCs differ markedly in biology. Single‐cell transcriptomic studies place DNs closer to developmentally arrested glutamatergic neuronal identities. BCs often show mixed astrocytic or undifferentiated progenitor‐like transcriptional profiles and are often electrophysiologically quiescent and may contribute to epileptogenesis primarily through indirect effects on the local microenvironment and circuitry.

## 3 Genetic Drivers: mTOR Pathway Somatic Mutations and Lesion Scale

### 3.1 Somatic Mutations Drive Neoplastic and Nonneoplastic Clonal Expansion in the Central Nervous System

During neural development and postmitotic life, endogenous DNA damage, imperfect repair, and transcription‐associated processes can generate somatic single‐nucleotide variants (SNVs). Using single‐neuron sequencing and model‐based correction, Lodato et al. estimated that neurons from the analyzed individuals may harbor approximately 1458–1580 somatic SNVs [17]. In this context, incidental hits on growth and metabolic regulatory networks are statistically plausible [18]. Most variants fail to meet the conditions for tissue‐level expansion or fixation as stable pathological phenotypes. Therefore, recurrent PI3K–AKT–mTOR activating mutations in FCD II are unlikely to represent mere background noise. Their pathological impact likely depends on developmental timing, cellular lineage, and local tissue conditions that allow mutant clones to persist or expand.

Clonal growth in FCD II diverges from the exponential expansion typical of malignancies. Affected lineages often leave the cell cycle late in cortical development or retain only limited proliferative capacity. Pathway activation more often produces enlarged cells, rewiring of metabolism and translation, and shifts in differentiation and migration. Vascular malformations, focal micro‐hypoxia, and neuroinflammation further sculpt lesion geometry and cellular composition, and tune local excitability thresholds. Clonal cells in FCD II are better pictured as developmental constraint coexisting with a pathological steady state.

### 3.2 Somatic mTOR Pathway Mutations in FCD II

**FIGURE 1:**
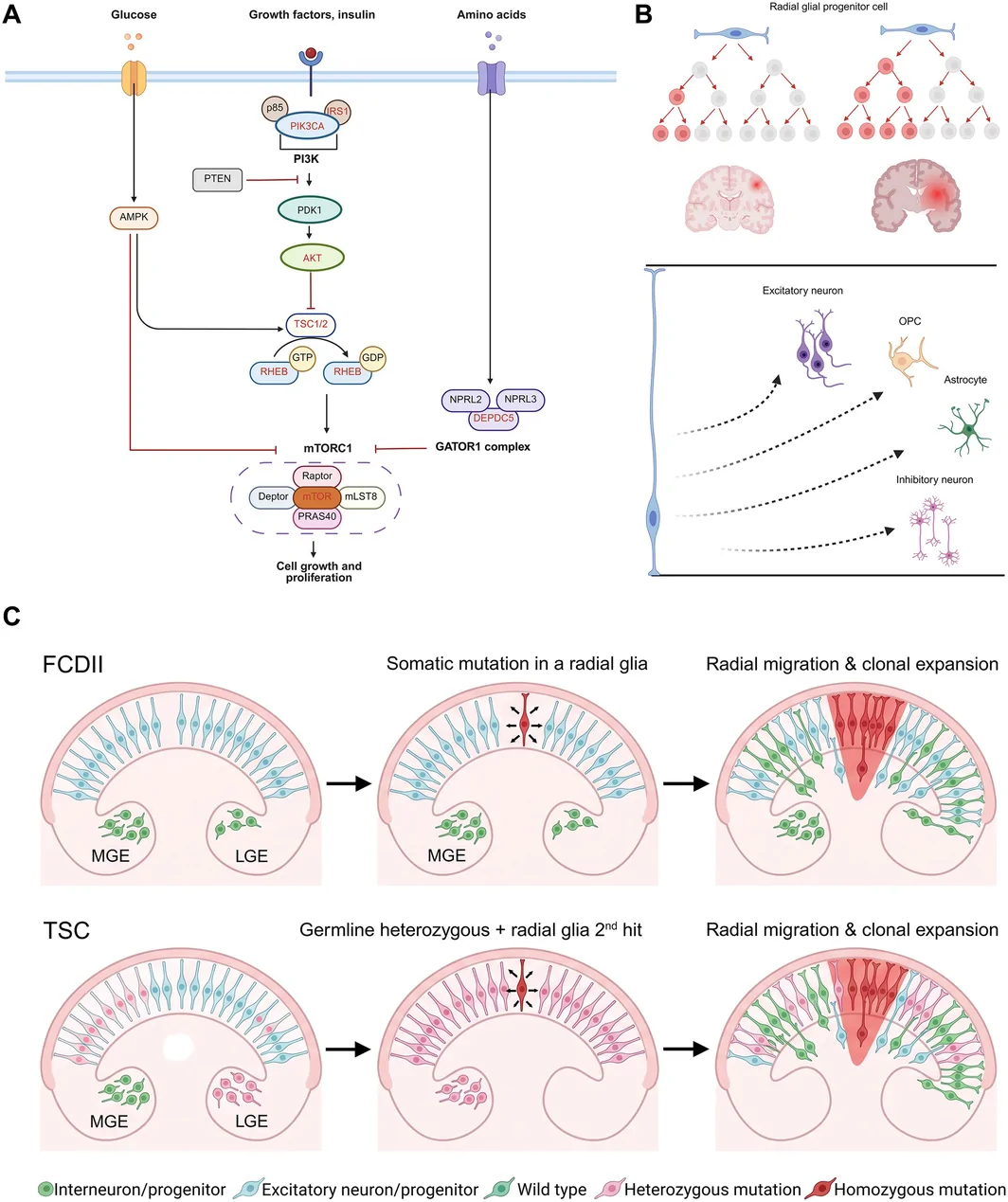
(A) Overview of the mTORC1 signaling pathway. Key inhibitors of mTOR signaling include *TSC1*, *TSC2*, *DEPDC5*, *NPRL2*, *NPRL3*, and *PTEN*, whereas activating variants in *PIK3CA*, *AKT3*, *RHEB*, or *MTOR* can increase pathway activity. (B) Developmental timing and lineage distribution of somatic variants shape lesion extent and cellular involvement in FCD II. Somatic variants arising in radial glial progenitor cells can be transmitted to their descendant cells during cortical development. Variants occurring earlier in development are expected to involve a larger progenitor clone and produce more extensive malformations, whereas later events affect fewer descendants and may give rise to smaller focal lesions. Depending on the developmental stage and lineage competence of the mutant progenitor, variant‐bearing cells may include excitatory neurons, oligodendrocyte precursor cells (OPCs), astrocytes, and inhibitory neurons. Red cells indicate variant‐bearing progeny; gray cells indicate nonmutant cells. (C) Schematic comparison of lesion genesis in FCD II versus TSC. In FCD II, a pathogenic somatic variant arising in a radial glial progenitor expands by radial migration to form a spatially restricted mutant clone (dark red) surrounded by wild‐type excitatory neurons (light blue) and wild‐type interneurons (green). Within this clone, aberrant mTORC1 activation is associated with focal cortical dyslamination and cytomegalic pathological cells (dysmorphic neurons ± balloon cells). In TSC, a constitutional heterozygous *TSC1* or *TSC2* variant (pink) creates a widespread one‐allele background; a subsequent somatic second hit in a radial glial progenitor then yields a localized biallelic focus (dark red) within otherwise heterozygous cortex, including heterozygous interneurons originating from the MGE and LGE. MGE, medial ganglionic eminence; LGE, lateral ganglionic eminence.

A substantial fraction of FCD II cases can be explained by somatic mosaic mutations that affect the PI3K‐AKT–mTOR pathway [9, 10, 11, 19, 20]. These variants are grouped into two mechanistic classes (Figure 1A). MTOR, PIK3CA, AKT3, and RHEB increase mTOR complex 1 (mTORC1) activity. TSC1 and TSC2 restrain mTORC1 primarily through inhibition of RHEB, whereas DEPDC5, as part of the GATOR1 complex [21, 22, 23, 24], negatively regulates amino acid–dependent mTORC1 activation through Rag GTPases. Their loss‐of‐function variants are also important. Consistent with pathway activation, increased phosphorylation of ribosomal protein S6 (pS6) is commonly used as a histological marker for FCD II [1]. Somatic variants in *IRS1* have been reported in FCD II with downstream ERK pathway activation rather than classic mTORC1 hyperphosphorylation [25]. Despite this, the mTOR pathway remains the predominant mechanistic driver in most FCD II cases.

Reported detection rates for mTOR‐related somatic variants fall roughly in the 30%–60% range. This interval is influenced by sampling site, sequencing depth, variant allele frequency (VAF) detection limits, and bioinformatic denoising [9]. In bulk surgical DNA, VAF approximates half the mosaic fraction (MF) for heterozygous autosomal somatic hits, so low‐VAF interpretation requires explicit depth thresholds and clone‐size reasoning [13, 26]. Absence of detected mutation should not be equated with absence of genetic drive. Some genetically unsolved cases may reflect detection limits below ~1% VAF as much as true pathway negativity. Deep sequencing of electrode‐adjacent surgical tissue may improve detection sensitivity compared with bulk sampling [27, 28, 29, 30].

### 3.3 Distribution of Somatic Mutations Across Cell Lineages

In resected FCD II tissue, somatic mutations are distributed across neuronal and glial lineages, and excitatory neurons often carry the main fraction of the pathological burden [11, 12]. In small lesions with VAF below ~5%, sorted surgical tissue suggests the pathogenic clone is enriched in neuronal rather than glial populations, whereas larger hemispheric lesions show broader neuronal–glial involvement [13].

### 3.4 Somatic Mutation Timing Shapes FCD Spectrum Disorders

Embryonic timing of mutation correlates with lesion scale. The earlier mutation occurs in neuroectodermal development, the higher the tissue mosaicism and the more extensive the cortical involvement tend to be. The somatic hit in most surgical cases occurs after establishment of forebrain founder progenitors [31, 32]. Hemimegalencephaly (HME) is thought to reflect earlier progenitor involvement, whereas smaller focal lesions may result from variants arising in later neural progenitor populations [13, 33, 34] (Figure 1B). Pathogenic mTOR variants in FCD II may show VAFs higher than expected for a spatially restricted late clone, a pattern that may reflect local enrichment or developmental expansion of mutant cell populations [9, 13]. HME provides an example of a malformation that is often associated with earlier and more widely distributed somatic variants [35]. This earlier, more widespread involvement can be related to the stage at which a somatic hit arises within the approximately 33 mitotic cycles of the neuroepithelium during corticogenesis [36]. This pattern explains why patients with the same pathway variant can differ markedly in clinical phenotype and EZ extent [37].

### 3.5 FCD II in the Landscape of mTORopathies

Tuberous sclerosis complex (TSC) and FCD II can be viewed as related mTORopathies within a shared framework of mTOR‐pathway dysregulation driven by developmental “hit” events [15]. In many sporadic FCD II cases, the prevailing model proposes a somatic activating mutation or two‐hit loss‐of‐function events affecting mTOR‐pathway repressors, arising in a neural progenitor within the developing cortex, generating a localized clonal patch of mutation‐bearing cells while surrounding tissue remains largely wild type (Figure 1C) [9, 15, 38]. By contrast, TSC is typically initiated by a heterozygous germline loss‐of‐function variant in *TSC1* or *TSC2*, so that cells across tissues, including interneuron lineages, carry one mutant allele [15, 39]. Cortical malformations in TSC are then proposed to emerge when a somatic second hit (or loss of heterozygosity) occurs within a progenitor lineage, producing a focal two‐hit lesion embedded within otherwise *TSC1*/*TSC2*‐heterozygous tissue (Figure 1C) [15, 39, 40]. Consistent with this shared mTOR‐centric mechanism, cortical lesions in both conditions can show aberrant mTORC1 activation and dysmorphic cells, contributing to epileptogenic potential [1, 14, 19]. TSC is nevertheless distinguished by wider multisystem involvement, frequent multifocal brain lesions, and constitutional heterozygosity that may contribute to broader neurodevelopmental phenotypes, including potential effects of interneuron haploinsufficiency [15, 41]. The presence of TORC1 activation and inflammatory signals in fetal TSC lesions suggests that inflammation may begin early and occur in parallel with cell‐autonomous mTOR hyperactivation [42]. Overall, comparison with TSC, together with the developmental timing spectrum linking FCD II to hemimegalencephaly [13, 33, 37], highlights how the timing and distribution of germline and somatic mTOR‐pathway alterations can yield overlapping yet clinically and pathologically distinct cortical malformations.

## 4 Cell‐Autonomous and Non‐Cell‐Autonomous Dual Mechanisms

### 4.1 Cell‐Autonomous and Non‐Cell‐Autonomous Mechanisms in Glutamatergic Neurons and Glia

**FIGURE 2:**
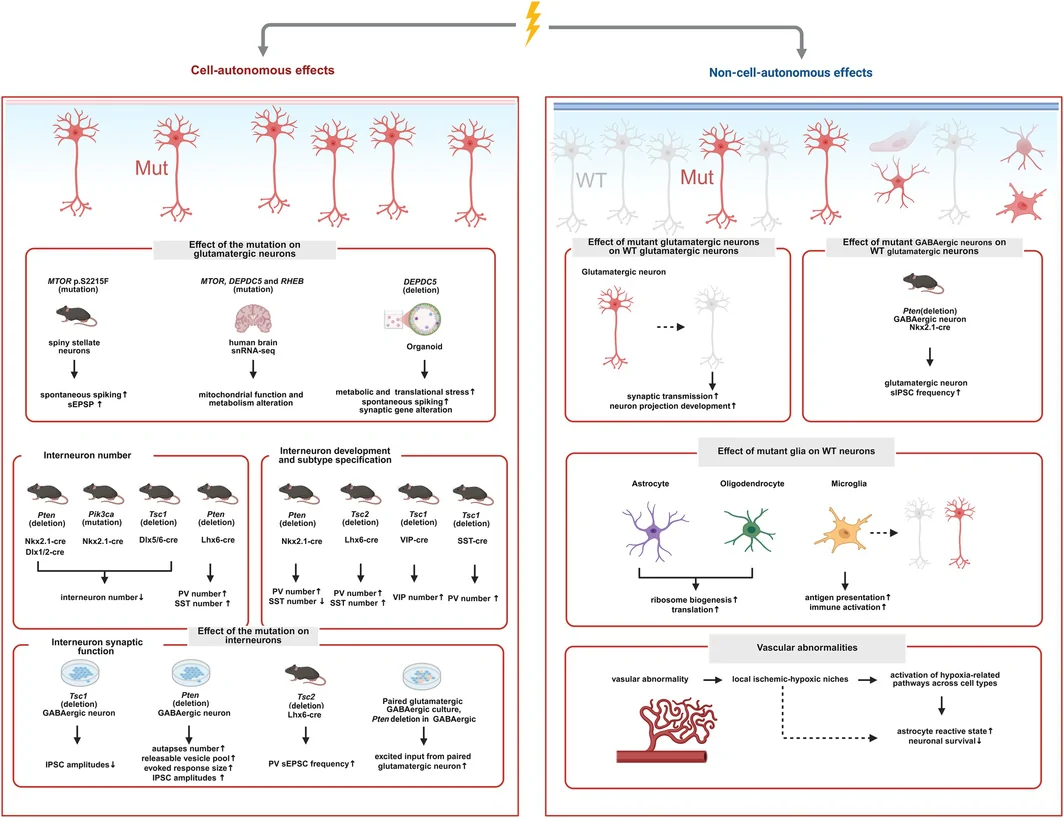
Cell‐autonomous and non‐cell‐autonomous mechanisms contributing to network dysfunction in FCD II. The schematic distinguishes intrinsic changes within mutation‐bearing cells from secondary effects on neighboring wild‐type cells and from lesion‐associated microenvironmental stress. Cell‐autonomous effects (left) refer to intrinsic consequences of somatic mTOR‐pathway mutations within mutant cells themselves, including altered morphology, metabolism, translation, excitability, and synaptic function in glutamatergic neurons and interneurons. Non‐cell‐autonomous effects (right) refer to secondary influences of mutation‐bearing cells on neighboring wild‐type cells: (1) effects of mutant glutamatergic neurons on wild‐type glutamatergic neurons; (2) effects of mutant GABAergic neurons on wild‐type glutamatergic neurons; and (3) effects of mutant glial cells (astrocytes, oligodendrocytes, and microglia) on neurons. Separately, vascular abnormalities can generate local ischemic–hypoxic niches that activate hypoxia‐related pathways across cell types, promote reactive astrocytic states, and compromise neuronal survival.

Single‐cell genotype studies suggest that mTOR mutations can trigger cell‐autonomous and non‐cell‐autonomous effects (Figure 2). On the cell‐autonomous aspect, mutant cells often become larger, experience metabolic stress, and show abnormal translation. Direct electrophysiological support for cell‐autonomous effects has been reported. In a mosaic rat model of FCD II carrying a recurrent *MTOR* p.S2215F somatic variant, neurons expressing mutant mTOR showed strong GluN2C‐containing NMDAR‐mediated spontaneous excitatory postsynaptic currents [43]. This translated into a higher rate of spontaneous spiking, but only during a narrow developmental period (P9–P20) [43]. Neighboring cells that were not electroporated (and therefore not mutant) did not show the same electrophysiological changes.

In contrast, non‐cell‐autonomous effects, likely communicated through signals in the microenvironment, may reshape synaptic function, axonal projections, and homeostatic programs in nearby nonmutant cells [11, 12, 32]. This operates through multifaceted mechanisms. First, mutant cells can actively reprogram neighboring nonmutant neurons and glia, inducing broad changes in synaptic and circuit‐level transcriptional programs. Notably, glutamatergic neurons lacking detectable mTOR variants (Ref.) still exhibit upregulation of synaptic transmission and axon/projection development pathways compared with age‐matched controls, with substantial overlap with variant‐carrying neurons (Mut.). Similar non‐cell‐autonomous remodeling is also observed in glial populations, indicating that seizure‐relevant pathways extend beyond mutation‐bearing cells [11]. Second, network dysfunction can emerge from the lesion microenvironment itself, independent of direct signaling from mutant cells. These include pathways related to microglial immune activation, synaptic homeostasis, and neuronal connectivity [12], which may disrupt the balance between excitatory neurons and inhibitory interneurons. Recent evidence highlights vascular malformations and abnormal smooth muscle derived “firework cells” that can impair cerebral blood flow and create localized ischemic–hypoxic niches, leading to widespread activation of hypoxia‐related pathways across cell types. Such conditions can alter astrocyte activity, induce reactive transcriptional states, and directly compromise neuronal survival, thereby contributing to network dysfunction beyond the mutation‐bearing cells. In addition, mutant astrocytes may further contribute by reshaping neuronal metabolic support, influencing synaptic remodeling, and modulating local ionic and neurotransmitter buffering. Importantly, these changes may reflect not only dysfunction but also compensatory adaptations, including enhanced metabolic support for energetically demanding mutant neurons and stabilization of a metabolically stressed microenvironment, thereby indirectly modulating neuronal state and network excitability.

### 4.2 Cell‐Autonomous and Non‐Cell‐Autonomous Mechanisms in Interneurons

mTOR pathway hyperactivation influences several aspects of interneuron development, including cell number, subtype specification, and synaptic function [44]. Evidence from genetic mouse models indicates that the effect depends strongly on the gene perturbed, the interneuron lineage targeted, and the developmental stage at which mTOR signaling is altered.

Several studies have shown that early activation of PI3K–mTOR signaling can reduce cortical interneuron numbers. Conditional deletion of *Pten* in interneuron progenitors using Nkx2.1‐Cre or Dlx1/2‐Cre decreased interneuron abundance [45]. A similar phenotype was observed when an active *Pik3ca* mutation was expressed in interneuron progenitors with Nkx2.1‐Cre. These mice also displayed reduced interneuron numbers [38]. Consistently, *Tsc1* deletion using Dlx5/6‐Cre, which targets intermediate interneuron progenitors, also lowered the number of cortical interneurons [46]. However, this relationship is not uniform across models. *Pten* deletion with Lhx6‐Cre resulted in an increase in both PV and SST interneurons [47]. These results probably reflect the stage‐specific and context‐dependent functions of mTOR signaling during interneuron generation, survival, and maturation [15].

mTOR hyperactivation can also alter interneuron subtype identity. *Pten* loss in Nkx2.1‐Cre‐defined interneuron progenitors biased interneuron production toward the PV lineage at the expense of SST interneurons [45]. When mTOR signaling was perturbed at a later developmental stage, *Tsc2* deletion using Lhx6‐Cre increased the density of interneurons co‐expressing PV and SST, again suggesting a shift of interneuron subtype identity [48]. Subtype‐restricted manipulations further support this idea. *Tsc1* deletion in VIP‐expressing interneurons using VIP‐Cre led to a relative increase in this population [49]. Likewise, *Tsc1* deletion with SST‐Cre increased the number of cells acquiring PV‐like properties [50]. Together, these studies indicate that mTOR signaling does not simply regulate interneuron abundance but also participates in determining interneuron phenotypic fate [15].

Beyond development and subtype specification, mTOR signaling has important roles in interneuron synaptic function. In single‐neuron culture systems, deletion of *Pten* from GABAergic neurons enhanced autaptic transmission, referring to synaptic connections formed by a neuron onto itself. This was associated with an increased number of autapses, enlargement of the readily releasable vesicle pool, and larger evoked responses [51]. However, subsequent work using postnatal Day 1‐equivalent single‐cell cultures showed that different routes to mTOR hyperactivation are not functionally equivalent: *Pten* loss increased inhibitory postsynaptic current amplitudes, whereas *Tsc1* loss reduced them [52]. In paired glutamatergic‐GABAergic neuron cultures, deleting *Pten* from the GABAergic neuron enhanced excitatory input from the paired glutamatergic neuron [53]. In vivo studies further support a role for mTOR in shaping interneuron synaptic function. Nkx2.1‐Cre‐mediated *Pten* deletion increased the frequency of spontaneous inhibitory postsynaptic currents onto excitatory cortical neurons [45]. Besides controlling inhibitory output from interneurons, mTOR can also modulate excitatory input to interneurons. For example, *Tsc2* deletion using Lhx6‐Cre elevated the density of excitatory (VGlut1+) synapses onto PV interneurons and the frequency of spontaneous excitatory postsynaptic currents [48]. Overall, these data suggest that mTOR dysregulation can reshape interneuron networks through multiple mechanisms, including altered interneuron production, subtype specification, survival, inhibitory output, and excitatory input [15].

## 5 DNs and BCs

DNs are a histological hallmark of FCD II, yet they constitute only a small fraction of lesional cells. Multimodal profiling and immunohistochemistry place many DNs closest to an aberrant glutamatergic state, characterized by high neurofilament expression, loss of canonical laminar markers, mitochondrial abnormalities, and alterations in synaptic gene programs with potential relevance to excitability [11]. In contrast, BCs occupy a distinct cellular state, with transcriptional profiles more closely aligned with astrocytes. Current evidence supports a lineage‐based model in which DNs and BCs represent distinct mutant populations arising from a shared somatic mutation acquired prior to the divergence of neuronal and astroglial lineages [11]. But this requires higher‐resolution spatiotemporal lineage tracing to further confirm.

Functionally, these two pathological cell populations are likely to engage the lesion through distinct mechanisms. DNs exhibit altered expression of genes related to neuronal excitability and synaptic function, including *SLC1A1*, and their altered synaptic machinery may directly influence the activity of neighboring nonmutant circuit elements. In contrast, BCs are enriched for secreted‐protein programs, such as *MFAP4* and *IGFBP7*, suggesting a predominantly paracrine mode of action on the local microenvironment and, consequently, indirect modulation of network states [11, 54].

Cytomegalic DNs and BCs together account for only approximately 1%–10% of mutation‐positive cells, indicating that the dysplastic cells represent only a minor fraction of the mutant clone [11]. A broader population of mutation‐bearing cells lacking classic cytomegalic morphology may nonetheless contribute to lesion formation through abnormal orientation, ectopic positioning, or altered physiological properties, thereby promoting dyslamination and network instability [11, 12]. These observations suggest that disease mechanisms cannot be fully explained by the most morphologically striking cell populations alone.

Electrophysiological studies further delineate the functional properties of these cells. Patch‐clamp recordings from resected cortical dysplasia tissue have demonstrated abnormal intrinsic membrane properties and synaptic responses in DNs, whereas many BCs appear electrically quiescent [55, 56, 57]. More recent work in Type II FCD shows that upper‐layer DNs fire at lower action‐potential frequencies and require larger current injections to reach firing threshold [58]. Although these findings establish that DNs are physiologically abnormal, they do not demonstrate that these cells are the primary drivers of the epileptic network [59]. Resolving this issue will require prospective studies linking cell‐type‐resolved physiological features with invasive epileptogenic‐zone mapping and postoperative seizure outcomes [60, 61]. Moreover, findings from FCD IIb further complicate a unified interpretation of DN identity, as a subset of DNs co‐express markers associated with inhibitory glycinergic signaling [62]. Whether DNs in FCD IIa and IIb share a common molecular identity or reflect subtype‐specific developmental and signaling states remains unresolved.

### 5.1 Speculative Framework: What May Distinguish FCD IIa From IIb?

FCD IIa and IIb are unlikely to be explained by any single genetic or developmental variable. We favor a view in which subtype identity reflects the joint contribution of (i) the strength and molecular route of mTOR‐pathway dysregulation and (ii) the developmental competence of the progenitor that acquires the somatic hit.

Both subtypes harbor DNs, so glutamatergic lineages are affected in either case. BCs in IIb add a further differentiation phenotype that is absent from IIa by definition. Genotype–phenotype mapping provides the clearest empirical foothold for the first dimension. In a deep histopathology series, loss‐of‐function variants in *GATOR1* genes (*DEPDC5*, *NPRL3*) were restricted to FCD IIa, whereas gain‐of‐function *MTOR* variants occurred in both IIa and IIb [63]. Multimodal single‐nucleus profiling has likewise placed cells with the highest mTORC1 pathway scores preferentially in IIb lesions [64]. These patterns are compatible with a preferential link between *MTOR* gain‐of‐function variants and BC–containing lesions, whereas *GATOR1* loss‐of‐function variants are enriched among FCD IIa cases without BCs in surgical series [63]. But this association should not be over‐interpreted. The same *MTOR* hotspot alleles, and MTOR‐pathway variants more broadly, can present as either IIa or IIb [9, 63, 65]. Focal embryonic *Depdc5* inactivation in mice can generate balloon‐like cells [66].

The second dimension concerns when and in which progenitor the hit arises. A mutation confined to a neuron‐committed lineage would be expected to yield mainly DNs and an IIa‐like histology. An earlier hit in a progenitor that still retains gliogenic potential could seed both neuronal and astroglial descendants and thereby permit BCs formation alongside DNs. Incomplete maturation along either lineage could also generate the mixed, immature phenotype associated with balloon cells, which co‐express neuronal, glial, and progenitor‐associated markers [67]. Single‐cell and spatial transcriptomics reinforce an astroglial‐leaning identity for BCs and a glutamatergic‐leaning identity for DNs, consistent with a shared somatic event before neuronal–astroglial divergence [11]. Transcriptomic neighborhood, however, does not by itself prove lineage history.

We therefore suggest that BC formation may require both ingredients at once: a sufficiently strong or qualitatively distinct pathway insult, and a progenitor that still retains broad developmental options. If only one of the two conditions is met, for example strong *MTOR* activation after neuronal commitment or a milder upstream lesion in an earlier progenitor, the lesion may still present as IIa. The same mTOR‐pathway genes can produce either subtype [9, 65, 68]. What may matter more is which cell is hit, at which developmental stage, and how the pathway is perturbed. This combined model remains a testable hypothesis and will need matched genotype, histology and lineage‐resolved assays in larger surgical series.

## 6 Tools for Dissecting DNs and BCs Mechanisms

In FCD II, BCs and DNs can be better understood using single‐cell multi‐omics. We review tools that combine RNA dynamics, chromatin‐age, and somatic genotyping to map pathological‐state emergence to lineage‐associated molecular trajectories.

### 6.1 Tools for Inferring Dysplastic Cell States and Trajectories in FCD IIb

Computational trajectory analyses require orthogonal experimental validation before being interpreted as developmental lineage relationships. RNA velocity and fate‐mapping approaches can infer directional cell‐state transitions and potential lineage fates from single‐cell transcriptomic data. Velocyto was the first widely used spliced/unspliced pipeline for RNA velocity [69]. scVelo extends this framework with improved steady‐state and dynamical models [69, 70]. Dynamo reconstructs absolute RNA velocity and continuous vector fields for fate prediction and in silico perturbation [71]. CellRank 2 converts velocity or expression kernels into fate probabilities and terminal‐state assignments [72]. RegVelo extends velocity with gene‐regulatory‐network priors to model regulation‐coupled dynamics and supports in silico perturbation [73]. Graph‐based, potency‐based, and latent‐space methods can reconstruct pseudotemporal ordering, branching trajectories, and developmental potential. Monocle 2 orders cells along reverse graph embedding trajectories [74]. Monocle 3 learns graph‐based pseudotime and branching trajectories in low‐dimensional embedding space. Slingshot fits branching principal curves on cluster minimum spanning trees [75]. PAGA abstracts cluster connectivity into a topology‐preserving graph for coarse lineage structure [76]. Palantir uses diffusion maps to assign fate probabilities along continuous differentiation [77]. scTour jointly infers pseudotime, vector fields and latent dynamics with a deep learning architecture [78]. CytoTRACE ranks relative plasticity from gene‐count heuristics [79]. CytoTRACE 2 adds interpretable deep‐learning potency categories on an absolute developmental scale [80]. Chromatin accessibility data can be used to estimate the mitotic or epigenetic age of individual cells. EpiTrace estimates mitotic age from scATAC‐seq clock loci [81].

### 6.2 Tools for Single‐Cell and Spatial Somatic Genotyping

Single‐cell RNA‐based genotyping can identify somatic variants in individual cells while linking genotype to cell‐type‐specific transcriptional profiles. Monopogen calls somatic SNVs directly from scRNA‐seq (optionally guided by matched scDNA) [82]. SComatic performs de novo somatic mutation detection from scRNA‐seq without matched normal DNA [83]. PRDD‐seq couples targeted clonal DNA variant sequencing with cell‐type‐informative RNA readouts in the same cells and provides an earlier template for linking barcoded clones to cortical cell classes [84]. Cell‐type‐informed genotyping workflows in mosaic focal epilepsy tissue assign pathway variants to excitatory neurons, glia, and other resident populations at scale [11, 12]. Spatial somatic mutation mapping can localize variant‐bearing clones within preserved tissue architecture. SpaceTracer detects somatic point mutations from spatial transcriptomics and aims to place variant‐bearing clones in intact tissue architecture [85].

## 7 Integrative Multi‐Omics Framework in FCD II

### 7.1 Surgical Tissue as the Integration Data Source

Resected cortex is the most critical data source for mechanistic and translational studies of drug‐resistant FCD II. A single operation can generate matched material for neuropathology, somatic variant detection, single‐cell or spatial profiling, germline comparison using blood‐derived DNA, and in patients undergoing invasive monitoring, electrode‐adjacent tissue that can be registered to clinically defined SOZ/EZ anatomy [2, 4, 9, 86]. Neuropathology identifies DNs, BCs in FCD IIb, and pathway‐activation markers such as pS6. Targeted sequencing, WES, and WGS can detect somatic PI3K–AKT–mTOR variants [1, 9, 10]. Single‐cell studies further show that DNs and BCs form only a minority of mutation‐positive cells [11]. What is still missing is a matched within‐patient chain from somatic genotype to spatial cell‐state map, ex vivo function, SOZ/EZ localization, and postoperative outcome.

### 7.2 Molecular and Spatial Profiling

Single‐cell and single‐nucleus approaches have reframed FCD II as more than a “mutant‐neuron‐only” disorder. Combined genotyping and transcriptomics can assign pathway variants to resident cell populations, including glutamatergic neurons, astrocytes, and oligodendroglia lineages, while distinguishing mutation‐bearing cells from transcriptionally altered reference cells within the same lesion [11, 12]. These data support both cell‐autonomous effects in mutant cells and non‐cell‐autonomous changes in neighboring cells, including synaptic, developmental, and glial programs that may extend epileptogenic influence beyond the genetically mutant clone.

Spatial multi‐omics approaches make it possible to resolve the spatial niches of FCD II in situ. These include spatial transcriptomics, spatial metabolomics, spatial proteomics, laser‐capture microdissection and pathology‐guided spatial panels. They reveal the molecular identity of DNs and BCs and capture how these cells influence the surrounding microenvironment. In doing so, they show how altered transcriptional, metabolic, and proteomic programs co‐localize with vascular, glial, and inflammatory microdomains [87].

### 7.3 Functional Validation

Functional studies are the necessary bridge between molecular pathology and epileptogenic mechanisms. As discussed above, patch‐clamp studies of resected FCD tissue have identified abnormal intrinsic and synaptic properties in DNs, whereas many BCs appear electrically quiescent [55, 56, 57]. Multielectrode arrays, organotypic cultures, and Patch‐seq could link network activity to molecularly defined cell types, but these approaches remain rarely applied to FCD II tissue. Patient‐derived cortical organoids and mosaic rodent models enable investigation of how defined mTOR‐pathway variants affect migration, morphology, synaptic transmission, and excitability [43, 88, 89].

### 7.4 Cross‐Modal Gaps and Translational Implications

An FCD II multi‐omics framework should connect the following levels: somatic variant, cell type or state, spatial microdomain, functional phenotype, SOZ/EZ context, and surgical outcome. Somatic variants can be detected in surgical tissue and assigned to cell populations. Spatial profiling can define dysplastic and microenvironmental niches. Ex vivo physiology can identify abnormal properties of dysmorphic neurons and altered excitation‐inhibition balance. SEEG and complete‐resection studies can relate SOZ/EZ anatomy to outcome [60, 61]. A key translational question is whether somatic variant burden and mTOR pathway activation are confined to the SOZ or extend into perilesional cortex that appears less severely affected on routine histology. In resected FCD II specimens, phospho‐S6 immunoreactivity and dysplastic histopathology provide tissue‐level evidence of mTOR‐pathway activation [1]. However, histopathological and pathway‐activation markers may not fully delineate the electrophysiologically defined epileptogenic zone [60, 61]. In a selected case, a mosaic *PIK3CA* variant showed a spatial gradient of variant allele fraction across SEEG‐defined regions [90]. In parallel, single‐cell genotyping and spatial profiling can localize variant‐bearing cell populations within dysplastic regions and their surrounding microenvironments [11, 12, 87].

Few studies, however, have combined these approaches within the same patient, with matched SEEG‐defined SOZ localization and systematic analysis of surgical margins. Moreover, seizure persistence after resection targeted to the MRI‐visible or histologically defined lesion indicates that the extent of the epileptogenic network may not be fully captured by conventional imaging or histopathology alone [2, 8]. This observation does not by itself establish that molecularly abnormal tissue extends beyond the resection margin, but it highlights the need to define the spatial relationship among mosaicism, mTOR activation, histopathological abnormalities, and electrophysiological epileptogenicity. These domains may overlap substantially in some patients but need not be congruent. Prospective studies integrating spatial somatic genotyping, pathway‐activation mapping, SEEG, surgical‐margin sampling, and postoperative outcomes are needed to determine whether molecular abnormalities beyond the resected lesion contribute to seizure recurrence [30, 91].

**FIGURE 3:**
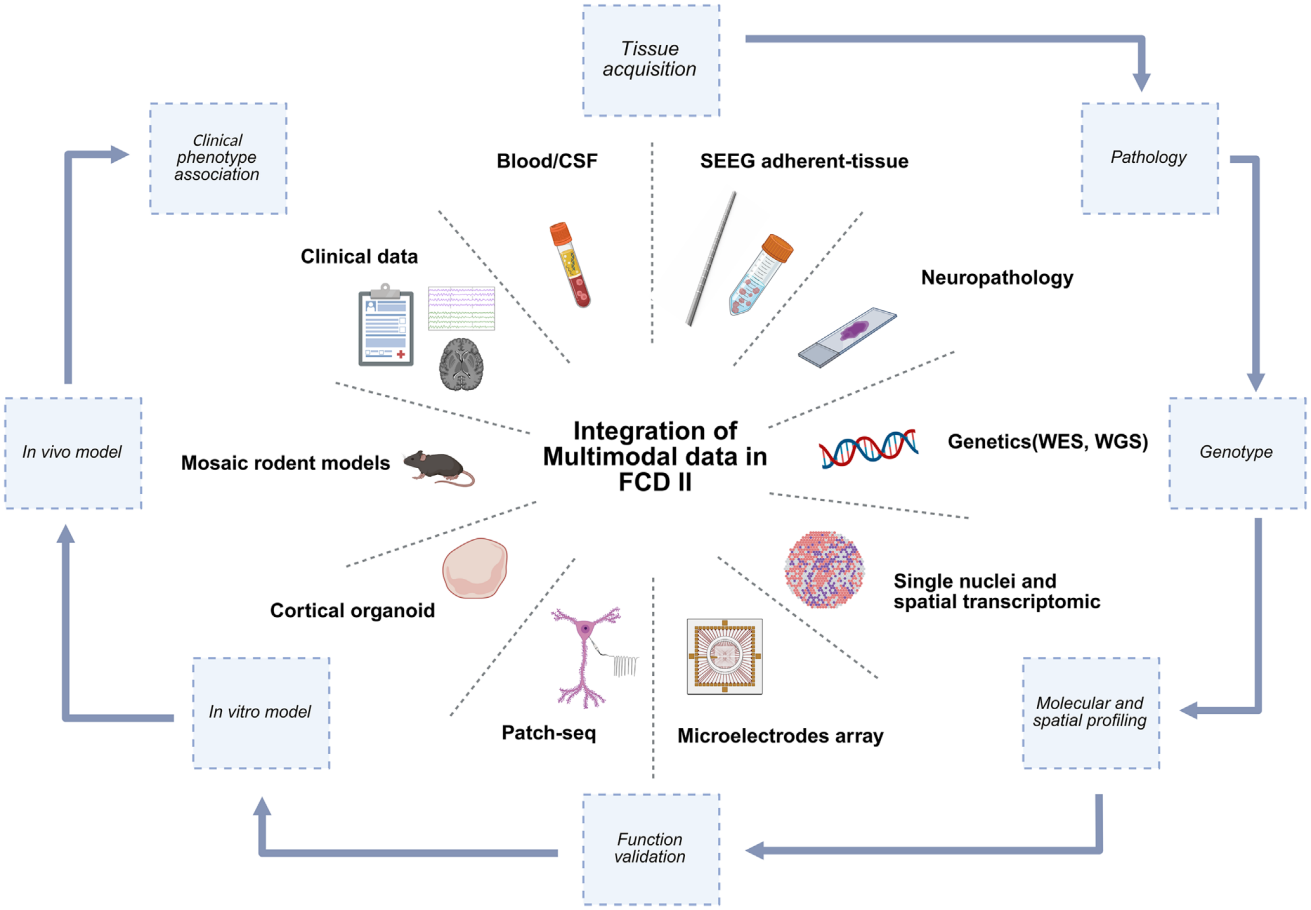
Integrative multimodal framework for FCD II. Surgical tissue serves as the central anchor for integrating clinical data, neuropathology, somatic genotyping, single‐nucleus and spatial profiling, and functional assays. Findings can be linked with SEEG‐defined epileptogenic regions, patient‐derived organoids, mosaic rodent models, and clinical phenotypes.

Multi‐omics can support mechanism‐guided stratification, but it does not yet provide a clinic‐ready treatment strategy. Three types of clinical interventions are plausible. First, genotype‐directed mTOR modulation is rational when a causal mosaic variant is confirmed. Second, microenvironment‐directed strategies targeting vascular, inflammatory, metabolic stress are suggested by spatial and cell‐state omics but remain mainly descriptive. Third, network‐level interventions, including antiseizure medication optimization, neuromodulation and tailored resection, remain clinically central. More exploratory approaches, including gene therapy, senolytics, and organoid‐informed drug screening, are also under investigation. The immediate priority is not a single biomarker, but prospectively matched multimodal cohorts that can test causal links across scales: genotype, cell state, spatial architecture, function, electrophysiological localization and surgical outcome [90, 91, 92] (Figure 3).

## 8 Conclusion

FCD II is increasingly understood as a developmental mosaic disorder. Somatic variants affecting the PI3K–AKT–mTOR pathway arise at different developmental stages and in different progenitor lineages, helping to explain variation in lesion size, cellular composition, and clinical presentation. Although DNs and BCs are central to pathological diagnosis, they represent only a small proportion of mutation‐positive cells. Other mutant cells, including those without overt cytomegaly, may also contribute to cortical disorganization and epileptogenicity. Seizure generation is likely to reflect both intrinsic abnormalities of mutant cells and changes in the surrounding tissue. Altered neuronal excitability and synaptic function may coexist with disrupted interneuron development, glial reactivity, vascular abnormalities, and local inflammatory or hypoxic responses. Their relative importance may depend on the causal variant, its timing and distribution, lesion subtype, and cortical location.

A major limitation of the field is the lack of studies that link these levels of evidence within individual patients. Future work should combine sensitive somatic variant detection with single‐cell and spatial profiling, tissue electrophysiology, SEEG‐based localization, and longitudinal surgical outcomes. Establishing these within‐patient links will help distinguish molecular correlates from mechanisms that directly contribute to epileptogenesis and may provide a basis for more meaningful biological stratification and targeted treatment of FCD II.

## Author Contributions

**Zesheng Li:** writing – original draft, visualization. **Yihe Wang:** visualization. **Jianwei Shi:** investigation. **Yumin Luo:** review and editing, supervision. **Yongzhi Shan:** resources, supervision. **Guoguang Zhao:** conceptualization, funding acquisition, supervision.

## Funding

This article received support from the Beijing Municipal Science & Technology Commission (Z221100007422016) and Beijing Natural Science Foundation‐Daxing Innovation Joint Fund (L246017).

## Conflicts of Interest

Yumin Luo is the Associate Editor of CNS Neuroscience and Therapeutics and a co‐author of this article. They were excluded from editorial decision‐making related to the acceptance and publication of this article. Editorial decision‐making was handled independently by the other editor to minimize bias.

## Data Availability

Data sharing not applicable to this article as no datasets were generated or analyzed during the current study.
